# Downregulation of Runx2 by 1,25-Dihydroxyvitamin D_3_ Induces the Transdifferentiation of Osteoblasts to Adipocytes

**DOI:** 10.3390/ijms17050770

**Published:** 2016-05-19

**Authors:** Jung Ha Kim, Semun Seong, Kabsun Kim, Inyoung Kim, Byung-Chul Jeong, Nacksung Kim

**Affiliations:** 1 Department of Pharmacology, Chonnam National University Medical School, Gwangju 61469, Korea; kjhpw@hanmail.net (J.H.K.); iamsemun@gmail.com (S.S.); kabsun@hanmail.net (K.K.); doll517@naver.com (I.K.); bcfrank9@gmail.com (B.-C.J.); 2 Department of Biomedical Sciences, Chonnam National University Medical School, Gwangju 61469, Korea

**Keywords:** 1,25(OH)_2_D_3_, transdifferentiation, osteoblast, adipocyte

## Abstract

1,25-Dihydroxyvitamin D_3_ (1,25(OH)_2_D_3_) indirectly stimulates bone formation, but little is known about its direct effect on bone formation. In this study, we observed that 1,25(OH)_2_D_3_ enhances adipocyte differentiation, but inhibits osteoblast differentiation during osteogenesis. The positive role of 1,25(OH)_2_D_3_ in adipocyte differentiation was confirmed when murine osteoblasts were cultured in adipogenic medium. Additionally, 1,25(OH)_2_D_3_ enhanced the expression of adipocyte marker genes, but inhibited the expression of osteoblast marker genes in osteoblasts. The inhibition of osteoblast differentiation and promotion of adipocyte differentiation mediated by 1,25(OH)_2_D_3_ were compensated by Runx2 overexpression. Our results suggest that 1,25(OH)_2_D_3_ induces the transdifferentiation of osteoblasts to adipocytes via *Runx2* downregulation in osteoblasts.

## 1. Introduction

Bone is an important organ for supporting the body and regulating mineral homeostasis. Bone is continuously remodeled by two cell types: osteoclasts and osteoblasts [1]. Osteoclasts resorb old bone and osteoblasts produce the bone matrix [2,3]. Various factors, such as the paratyroid hormone and 1,25-dihydroxyvitamin D_3_ (1,25(OH)_2_D_3_), regulate bone homeostasis by modulating bone cells [2,4].

The steroid hormone 1,25(OH)_2_D_3_ is a key regulator of calcium homeostasis and skeletal health [5]. In bone, 1,25(OH)_2_D_3_ regulates mineralization both in an indirect and a direct manner. 1,25(OH)_2_D_3_ increases calcium absorption from the intestines, which indirectly stimulates bone formation [6]. It can also directly influence bone formation via the regulation of osteoblast differentiation and function. Various studies have demonstrated the direct effects of 1,25(OH)_2_D_3_ on osteoblast differentiation and function *in vitro*. However, the results of these studies are highly controversial. Several studies have shown that 1,25(OH)_2_D_3_ stimulates osteoblast differentiation by increasing alkaline phosphatase (ALP) activity and osteocalcin expression in human primary osteoblast-like cells [7,8,9]. However, several lines of evidence suggest that 1,25(OH)_2_D_3_ negatively regulates osteoblast differentiation. For example, 1,25(OH)_2_D_3_ downregulates osteocalcin expression in mouse calvaria 3T3 (MC3T3)-E1 osteoblasts [10]. Lieben *et al.* [11] have also reported that 1,25(OH)_2_D_3_ impairs mineralization by upregulating the expression of mineralization inhibitors in mouse primary osteoblasts.

Osteoblasts, which are responsible for bone formation, are derived from mesenchymal stem cells by the action of several transcription factors, including Runx2, osterix, and β-catenin [12]. In particular, Runx2, a cell-specific member of the Runt family of transcription factors, is essential for mesenchymal cell differentiation into osteoblasts. Runx2 promotes the acquisition of an osteoblastic phenotype by mesenchymal stem cells by inducing the expression of genes encoding major bone matrix proteins, e.g., *Col1a1*, osteopontin, bone sialoprotein (*BSP*), and osteocalcin [11,12]. Runx2^−/−^ mice exhibit a complete lack of intramembranous and endochondral ossification *in vivo* and Runx2^−/−^ calvarial cells cannot differentiate into osteoblasts, even in the presence of osteogenic factors *in vitro* [12,13,14]. Multiple factors that play an important role in osteoblast differentiation via the regulation of Runx2 expression or activation have been identified.

Adipocytes as well as osteoblasts are derived from mesenchymal stem cells. Various transcription factors, such as CCAAT/enhancer binding protein-α (CEBP-α), CEBP-β, and peroxisome proliferator-activated receptor-γ (PPAR-γ), are essential for mesenchymal cell differentiation into adipocytes [15]. Increased adipose tissue in the bone marrow of osteoporotic patients and individuals with age-dependent bone loss may be associated with the transdifferentiation of osteoblasts to adipocytes [16]. While the effect of 1,25(OH)_2_D_3_ on the transdifferentiation of skeletal muscle cells to adipose cells is known, it is not clear if 1,25(OH)_2_D_3_ is involved in the transdifferentiation of osteoblasts to adipocytes in the bone marrow [17].

To elucidate the direct effect of locally produced 1,25(OH)_2_D_3_ on bone formation, we investigated the effect of 1,25(OH)_2_D_3_ on osteoblast differentiation, *Runx2* expression, and the transdifferentiation of osteoblasts to adipocytes.

## 2. Results

### 2.1. 1,25-Dihydroxyvitamin D_3_ (1,25(OH)_2_D_3_) Inhibits Osteoblast Differentiation and Runx2 Expression in Primary Osteoblasts

**Figure 1:**
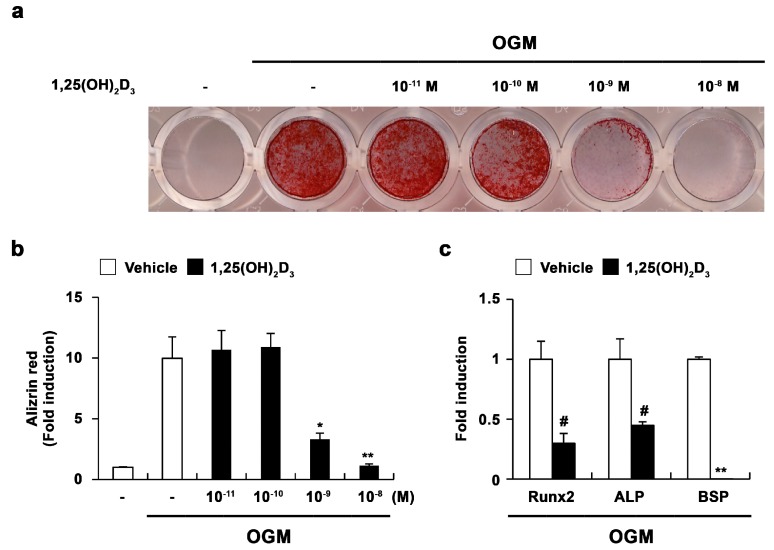
1,25-dihydroxyvitamin D_3_ (1,25(OH)_2_D_3_) inhibits osteoblast differentiation. (**a**–**c**) Primary osteoblasts were cultured in osteogenic medium (OGM) with vehicle or increasing concentrations of 1,25(OH)_2_D_3_. (**a**) Cultured cells were fixed and stained with Alizarin red; (**b**) Alizarin red staining was quantified by densitometry at 562 nm; (**c**) Primary osteoblasts were cultured in osteogenic medium (OGM) containing either vehicle or 1,25(OH)_2_D_3_ (10^−8^ M). The mRNA levels of *Runx2*, alkaline phosphatase (*ALP*), and bone sialoprotein (*BSP*) were analyzed by real-time polymerase chain reaction (PCR). # *p* < 0.05; * *p* < 0.01; ** *p* < 0.001 as compared with controls.

To elucidate the direct effect of 1,25(OH)_2_D_3_ on bone formation, we examined its role in osteoblast differentiation. When mouse primary osteoblasts were cultured in osteogenic medium including bone morphogenic protein 2 (BMP2), ascorbic acid, and β-glycerophosphate, bone nodule formation was dramatically induced (Figure 1a,b). However, supplementation with 1,25(OH)_2_D_3_ significantly inhibited osteoblast differentiation induced by the osteogenic medium in a dose-dependent manner (Figure 1a,b). The negative effect of 1,25(OH)_2_D_3_ on osteogenesis was confirmed by the expression of osteoblast differentiation-related genes. As shown in Figure 1c, 1,25(OH)_2_D_3_ inhibited the expression of osteoblastic genes, such as *Runx2*, *ALP*, and *BSP*. Therefore, 1,25(OH)_2_D_3_ may negatively regulate osteoblast differentiation by inhibiting the expression of *Runx2* and its downstream targets *ALP* and *BSP*.

### 2.2. 1,25(OH)_2_D_3_ Induces the Transdifferentiation of Osteoblasts to Adipocytes

**Figure 2:**
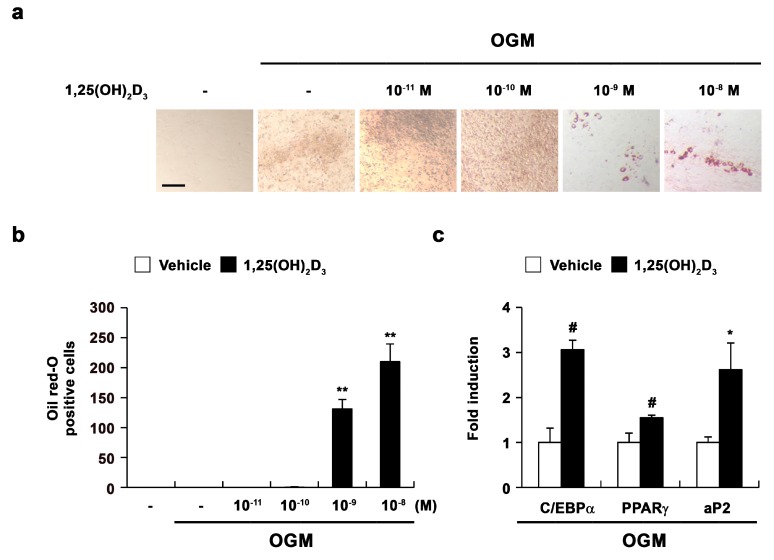
1,25(OH)_2_D_3_ induces transdifferentiation of osteoblasts to adipocytes. (**a**–**c**) Primary osteoblasts were cultured in osteogenic medium (OGM) with vehicle or increasing concentrations of 1,25(OH)_2_D_3_. (**a**) Cultured cells were fixed and stained with Oil Red-O, Bar: 50 µm; (**b**) The number of Oil Red-O—positive cells was counted; (**c**) Primary osteoblasts were cultured in osteogenic medium (OGM) containing either vehicle or 1,25(OH)_2_D_3_ (10^−8^ M). The mRNA levels of CCAAT/enhancer binding protein-α (*CEBP-α*), peroxisome proliferator-activated receptor-γ (*PPAR-γ*), and adipocyte protein 2 (*aP*) were analyzed by real-time PCR. # *p* < 0.05; * *p* < 0.01; ** *p* < 0.001 as compared with controls.

Intriguingly, 1,25(OH)_2_D_3_ treatment during osteoblast differentiation resulted in lipid droplet formation with the inhibition of bone nodule formation. Therefore, we examined the effect of 1,25(OH)_2_D_3_ on adipocyte differentiation during osteoblast differentiation. Osteoblasts were cultured in osteogenic medium either without or with 1,25(OH)_2_D_3_. Positive Oil Red-O staining confirmed the presence of lipid droplets, which were primarily observed in osteoblasts treated with high concentrations of 1,25(OH)_2_D_3_ (Figure 2a,b). Additionally, 1,25(OH)_2_D_3_ significantly increased the expression of adipocyte marker genes, including *CEBP*-α, *PPAR*-γ, and adipocyte protein 2 (*aP2*), compared with control cells. These results indicated that 1,25(OH)_2_D_3_ can induce the transdifferentiation of osteoblasts to adipocytes during osteoblast differentiation.

**Figure 3:**
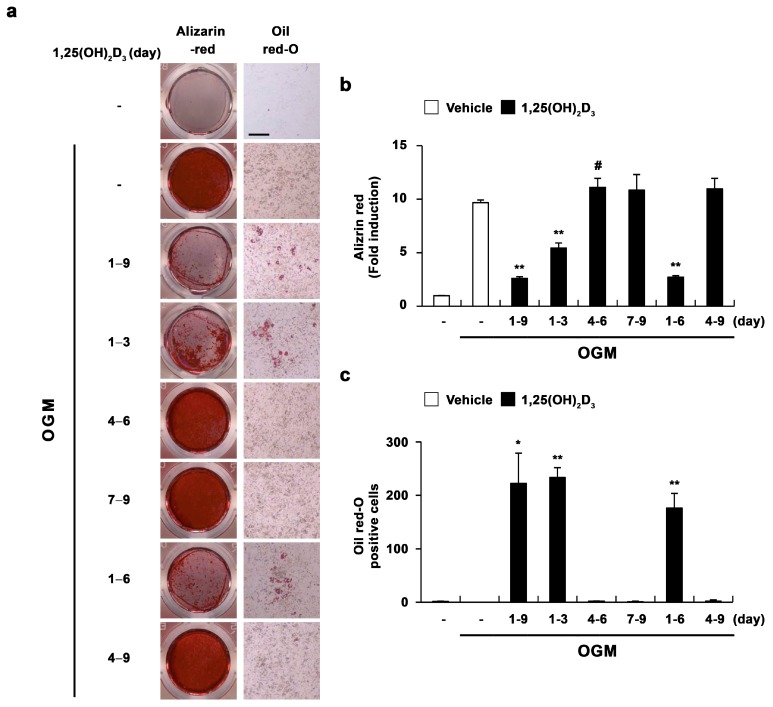
1,25(OH)_2_D_3_ induces the transdifferentiation of osteoblasts to adipocytes at the early stage of osteoblast differentiation. (**a**–**c**) Primary osteoblasts were cultured in osteogenic medium (OGM). Cells were treated with either vehicle or 1,25(OH)_2_D_3_ (10^−8^ M) during the indicated time periods. (**a**) Cultured cells were fixed and stained with Alizarin red or Oil Red-O, Bar: 50 µm; (**b**) Alizarin red staining was quantified by densitometry at 562 nm; (**c**) The number of Oil Red-O—positive cells was counted. # *p* < 0.05; * *p* < 0.01; ** *p* < 0.001 as compared with controls.

Next, we assessed the time course of the effect of 1,25(OH)_2_D_3_ on the transdifferentiation of osteoblasts to adipocytes. When 1,25(OH)_2_D_3_ was added continuously from the start of osteoblast differentiation (days 1–9), adipocyte differentiation was dramatically enhanced, while osteoblast differentiation was significantly reduced (Figure 3). Only the effect of 1,25(OH)_2_D_3_ added from day 1 to day 6 was comparable to that of 1,25(OH)_2_D_3_ added continuously from the start of osteoblast differentiation (*i.e.*, days 1–9) (Figure 3). However, the addition of 1,25(OH)_2_D_3_ during the late stage of osteoblast differentiation (days 7–9) did not affect the transdifferentiation of osteoblasts to adipocytes. Interestingly, the effect of 1,25(OH)_2_D_3_ on the transdifferentiation of osteoblasts to adipocytes was dependent on the addition of 1,25(OH)_2_D_3_ during the initial three days of osteoblast differentiation (Figure 3). Taken together, these results demonstrated that 1,25(OH)_2_D_3_ primarily acts at the early stage of osteoblast differentiation to induce the transdifferentiation of osteoblasts to adipocytes.

### 2.3. 1,25(OH)_2_D_3_ Induces Adipocyte Differentiation in Osteoblasts

**Figure 4:**
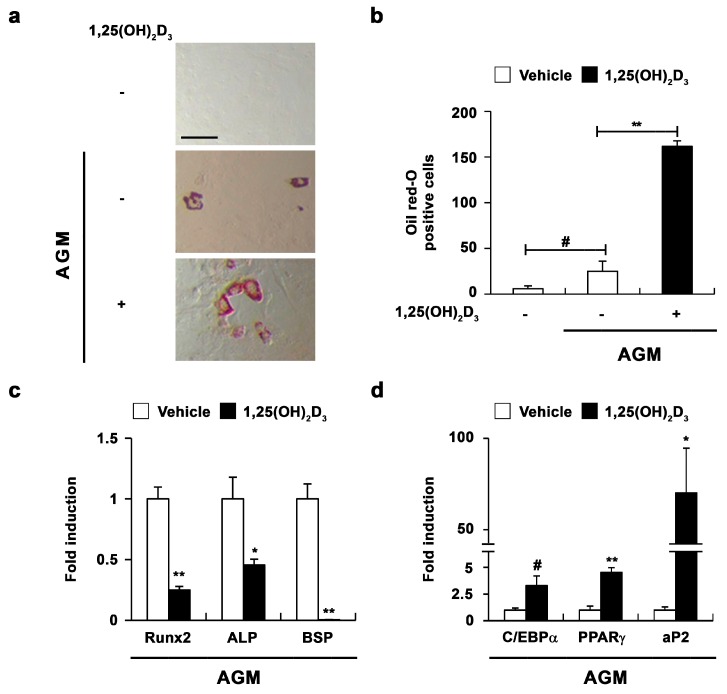
1,25(OH)_2_D_3_ induces adipocyte differentiation in primary osteoblasts. (**a**–**d**) Primary osteoblasts were cultured in adipogenic medium (AGM) containing either vehicle or 1,25(OH)_2_D_3_ (10^−8^ M). (**a**) Cultured cells were fixed and stained with Oil Red-O, Bar: 50 µm; (**b**) The number of Oil Red-O—positive cells was counted; (**c**) The mRNA levels of *Runx2*, *ALP*, and *BSP* were analyzed by real-time PCR; (**d**) The mRNA levels of *CEBP*-α, *PPAR*-γ, and *aP2* were analyzed by real-time PCR. # *p* < 0.05; * *p* < 0.01; ** *p* < 0.001 as compared with controls.

To confirm the role of 1,25(OH)_2_D_3_ in osteoblasts, we investigated the effect of 1,25(OH)_2_D_3_ during adipocyte differentiation of osteoblasts. When mouse primary osteoblasts were cultured in adipogenic medium including insulin, dexamethasone, 3-isobutyl-1-methylxanthine (IBMX), and rosiglitazone, the formation of lipid droplets was slightly induced (Figure 4a,b). As shown in Figure 4, 1,25(OH)_2_D_3_ significantly enhanced lipid droplet formation induced by adipogenic medium (Figure 4a,b). Additionally, 1,25(OH)_2_D_3_ increased the expression of adipocyte marker genes, but decreased the expression of osteoblast marker genes (Figure 4c,d). These results suggested that 1,25(OH)_2_D_3_ regulates not only osteoblast differentiation, but also adipocyte differentiation in osteoblasts.

### 2.4. 1,25(OH)_2_D_3_ Induces the Transdifferentiation of Osteoblasts to Adipocytes via the Regulation of Runx2 Expression

**Figure 5:**
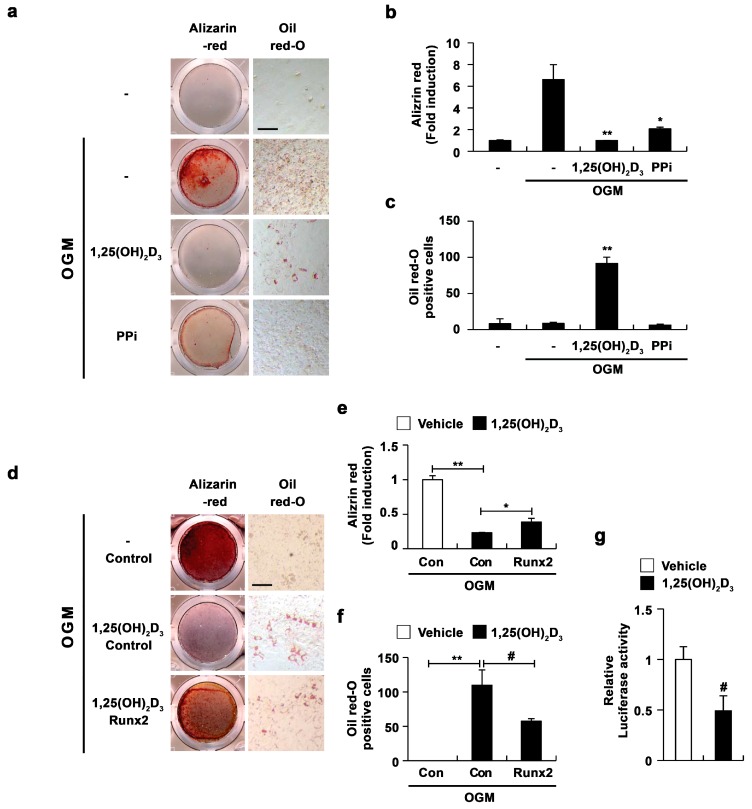
1,25(OH)_2_D_3_ induces transdifferentiation of osteoblasts to adipocytes by inhibiting *Runx2* expression. (**a**–**c**) Primary osteoblasts were cultured in osteogenic medium (OGM) with vehicle, 1,25(OH)_2_D_3_, or PPi as indicated. (**a**) Cultured cells were fixed and stained with Alizarin red or Oil Red-O, Bar: 50 µm; (**b**) Alizarin red staining was quantified by densitometry at 562 nm; (**c**) The number of Oil Red-O–positive cells was counted; (**d**–**f**) Osteoblasts were transduced with pMX-IRES-*EGFP* (control) or Runx2 retrovirus. Transduced osteoblasts were cultured in osteogenic medium (OGM) with vehicle or 1,25(OH)_2_D_3_. (**d**) Cultured cells were fixed and stained with Alizarin red or Oil Red-O, Bar: 50 µm; (**e**) Alizarin red staining was quantified by densitometry at 562 nm; (**f**) The number of Oil Red-O—positive cells was counted; (**g**) C2C12 cells were transfected with a *Runx2* reporter construct. Transfected cells were treated with vehicle or 1,25(OH)_2_D_3_ for 48 h. Luciferase activity was measured using a dual-luciferase reporter assay system. The data represent means and SD of triplicate samples. # *p* < 0.05; * *p* < 0.01; ** *p* < 0.001 as compared with controls.

It has recently been reported that 1,25(OH)_2_D_3_ mediates the suppression of mineral incorporation via the upregulation of pyrophosphate (PPi) levels [11]; accordingly, we analyzed whether increased PPi levels due to 1,25(OH)_2_D_3_ are involved in the transdifferentiation of osteoblasts to adipocytes. The addition of PPi to cultured osteoblasts suppressed osteoblast differentiation, similar to treatment with 1,25(OH)_2_D_3_ (Figure 5a,b). However, PPi did not induce the formation of lipid droplets during osteoblastogenesis (Figure 5a,c). Therefore, these results indicated that increased PPi levels in response to 1,25(OH)_2_D_3_ are involved in the inhibitory effect of 1,25(OH)_2_D_3_ on mineralization, but not in the stimulatory effect of 1,25(OH)_2_D_3_ on the formation of lipid droplets.

Since 1,25(OH)_2_D_3_ inhibited the expression of *Runx2* (Figure 1c), we next analyzed whether the inhibitory effect of 1,25(OH)_2_D_3_ on *Runx2* expression regulates adipocyte differentiation during osteoblast differentiation. The overexpression of *Runx2* restored the decrease in osteoblast differentiation as well as the increase in adipocyte differentiation regulated by 1,25(OH)_2_D_3_ (Figure 5d,f). Moreover, the inhibitory effect of 1,25(OH)_2_D_3_ on *Runx2* expression was confirmed by the 1,25(OH)_2_D_3_-mediated suppression of *Runx2* promoter activity (Figure 5g). Taken together, these results demonstrated that the downregulation of *Runx2* by 1,25(OH)_2_D_3_ in osteoblasts results in the inhibition of osteoblast differentiation accompanied by the transdifferentiation of osteoblasts to adipocytes. However, because *Runx2* overexpression did not completely reverse the effect of 1,25(OH)_2_D_3_ on adipocyte and osteoblast differentiation, there remains a possibility that 1,25(OH)_2_D_3_ regulates the transdifferentiation of osteoblasts to adipocytes through unknown pathways other than *Runx2* regulation.

## 3. Discussion

It is generally accepted that 1,25(OH)_2_D_3_ stimulates bone formation by increasing calcium absorption from the intestines [6]. However, the role of 1,25(OH)_2_D_3_ in bone formation mediated by osteoblasts is still largely unclear. Here, we showed that 1,25(OH)_2_D_3_ directly suppressed osteoblast differentiation, potentially as a result of *Runx2* inhibition.

Runx2 is a key transcription factor that initiates and regulates the early stage of osteoblast differentiation. An inhibitory effect of 1,25(OH)_2_D_3_ on osteoblast differentiation was observed when 1,25(OH)_2_D_3_ was added at the early stage, rather than the late stage of osteoblast differentiation. Furthermore, 1,25(OH)_2_D_3_ suppressed the expression of *Runx2* during osteoblast differentiation, which in turn inhibited the expression of downstream genes, such as *ALP* and *BSP*. Similarly, it has been reported that 1,25(OH)_2_D_3_ suppresses *Runx2* expression within 24 h in MC3T3 and rat osteosarcoma (ROS) 17/2.8 cells via the binding of vitamin D receptor (VDR) to vitamin D response element (VDRE) and Runx2 autonomously suppresses its own expression [18]. In contrast, Han *et al.* [19] reported that 1,25(OH)_2_D_3_ increases Runx2 expression in vascular smooth muscle cells and induces vascular calcification. These findings suggest that 1,25(OH)_2_D_3_ inhibits bone formation by suppressing Runx2 expression in osteoblasts, but the effect of 1,25(OH)_2_D_3_ on bone mineralization may be dependent on the cell-type specificity of Runx2 expression regulated by 1,25(OH)_2_D_3_.

The direct effect of 1,25(OH)_2_D_3_ on adipocyte differentiation is not yet clear. Several studies have reported a negative role of 1,25(OH)_2_D_3_ in adipogenesis [20,21,22]. However, it has also been reported that 1,25(OH)_2_D_3_ promotes the maturation of human subcutaneous preadipocytes via a PPAR-γ-independent pathway [23]. Previous studies have indicated that 1,25(OH)_2_D_3_ does not directly induce critical early factors for adipocyte differentiation. 1,25(OH)_2_D_3_-mediated inhibition of osteoblast differentiation accompanied by an increase in lipid droplet formation suggests an important role of 1,25(OH)_2_D_3_ in the transdifferentiation of osteoblasts to adipocytes. Previous results have also shown that 1,25(OH)_2_D_3_ can induce the formation of lipid droplets in osteoblasts [24]. Similarly, we observed that 1,25(OH)_2_D_3_ stimulated the formation of lipid droplets, even in osteogenic medium that lacked adipogenic stimulators. The formation of lipid droplets in osteoblasts was more highly stimulated in 1,25(OH)_2_D_3_-containing osteogenic medium than in adipogenic medium including insulin, dexamethasone, IBMX, and rosiglitazone. Therefore, the transdifferentiation of osteoblasts to adipocytes induced by 1,25(OH)_2_D_3_ is involved in the regulation of osteoblastic genes, rather than the induction of adipogenic genes. Runx2 inhibits the late adipocyte maturation of human bone marrow precursor cells and Runx2-deficient osteoblasts spontaneously undergo adipocyte differentiation with the inhibition of osteoblast differentiation [25,26,27]. In our study, 1,25(OH)_2_D_3_ inhibited *Runx2* expression and the overexpression of *Runx2* suppressed the formation of lipid droplets induced by 1,25(OH)_2_D_3_, suggesting that Runx2 downregulation mediated by 1,25(OH)_2_D_3_ induces the transdifferentiation of osteoblasts to adipocytes. Recently, it has been reported that adipocytes support osteoclast differentiation and function via receptor activator of nuclear factor kappa-B ligand (RANKL) production [28]. It is also well known that 1,25(OH)_2_D_3_ indirectly induces osteoclast differentiation via RANKL upregulation in osteoblasts [29,30]. 1,25(OH)_2_D_3_ may primarily enhance RANKL in osteoblasts and secondarily enhance RANKL by increasing adipocyte differentiation. Although vitamin D supplementation indirectly increases bone mass by improving intestinal calcium absorption, the administration of high-dose vitamin D to older woman results in an increased fracture risk [31,32]. Vitamin D might have several roles in osteoblasts, including the inhibition of osteoblast differentiation, promotion of adipocyte differentiation, and the support of osteoclast differentiation, and these functions may result in unexpected deleterious effects of vitamin D on the skeleton.

## 4. Materials and Methods

### 4.1. Reagents

Recombinant human BMP2 was purchased from Cowellmedi (Busan, Korea). Alizarin red, β-glycerophosphate, insulin, dexamethasone, 3-isobytyl-1-methylxanthine, Oil Red-O, rosiglitazone, and 1,25(OH)_2_D_3_ were purchased from Sigma-Aldrich (St. Louis, MO, USA). Ascorbic acid was purchased from Junsei Chemical (Tokyo, Japan).

### 4.2. Osteoblast Differentiation

Primary osteoblast precursor cells were isolated from neonatal mouse calvaria by digestion with 0.1% collagenase (Life Technologies, Carlsbad, CA, USA) and 0.2% dispase II (Roche Diagnostics GmbH, Mannheim, Germany). Isolated osteoblast precursor cells were cultured in α-Minimal Essential Medium containing 10% fetal bovine serum, 100 U/mL penicillin, and 100 mg/mL streptomycin. For osteoblast differentiation, primary osteoblast precursor cells were cultured in osteogenic medium containing BMP2 (100 ng/mL), ascorbic acid (50 µg/mL), and β-glycerophosphate (100 mM) for six to nine days. Cultured cells were fixed with 70% ethanol and stained with 40 mM alizarin red (pH 4.2). After nonspecific staining was removed with phosphate-buffered saline, alizarin red staining was visualized with a CanoScan 4400F (Canon Inc., Tokyo, Japan). Alizarin red-stained cells were dissolved with 10% cetylpyridinium (Sigma-Aldrich), and absorbance of the extracted solution was measured at 562 nm for quantification.

### 4.3. Adipocyte Differentiation

Primary osteoblast precursor cells were cultured in adipogenic medium containing insulin (1 µg/mL), dexamethasone (0.1 µM), rosiglitazone (1 µM), and 3-isobytyl-1-methylxanthine (0.5 mM) for two days. Cells were further cultured in adipogenic medium containing insulin (1 µg/mL) for six to nine days. Cultured cells were fixed with 3.7% formalin and stained with Oil Red-O solution.

### 4.4. Real-Time Polymerase Chain Reaction (PCR)

Quantitative real-time PCR analyses were performed as previously described [33,34]. Each analysis was performed in triplicate with a Rotor-Gene Q (Qiagen, Hilden, Germany) using SYBR green mixture (Qiagen). Target gene expression was normalized to the levels of an endogenous control, *i.e.*, glyceraldehyde-3-phosphate dehydrogenase (*Gapdh*). The relative quantitative value of each target gene compared to the calibrator for that target was expressed as 2^−(*C*t−*C*c)^, wherein *C*_t_ and *C*c represent the mean threshold cycle differences after normalization to *Gapdh*. The relative expression levels of samples are presented on a semi-log scale. The following oligonucleotide primers were used for real-time PCR: *Gapdh*, 5′-TGACCACAGTCCATGCCATCACTG-3′ and 5′-CAGGAGACAACCTGGTCCTCAGTG-3′; *Runx2*, 5′-CCCAGCCACCTTTACCTACA-3′ and 5′-CAGCGTCAACACCATCATTC-3′; *Alp*, 5′-CAAGGATATCGACGTGATCATG-3′ and 5′-GTCAGTCAGGTTGTTCCGATTC-3′; *BSP*, 5′-GGAAGAGGAGACTTCAAACGAAG-3′ and 5′-CATCCACTTCTGCTTCTTCGTTC-3′; *C/EBP*α, 5′-AAGAAGTCGGTGGACAAGAACAG-3′ and 5′-TGCGCACCGCGATGT-3′; *PPAR*γ, 5′-TCCAGCATTTCTGCTCCACA-3′ and 5′-ACAGACTCGGCACTCAATGG-3′; *aP2*, 5′-AAATCACCGCAGACGACA-3′ and 5′-CACATTCCACCACCAGCT-3′.

### 4.5. Luciferase Assay

Mouse myoblasts C2C12 cells were plated in 24-well plates at a density of 2 × 10^4^ cells/well one day before transfection. The *Runx2* reporter plasmid was transfected into C2C12 cells using Attractene according to the manufacturer’s instructions. Transfected cells were treated with vehicle or 1,25(OH)_2_D_3_ (10^−8^ M) for two days. Luciferase activity was measured using a dual-luciferase reporter assay system (Promega, Madison, WI, USA) according to the manufacturer’s instructions. 

### 4.6. Retroviral Gene Transduction

The retrovirus packaging cell line Plat-E was maintained in Dulbecco’s modified Eagle’s medium supplemented with 10% fetal bovine serum, puromycin (1 µg/mL), and blasticidin (10 µg/mL). To obtain viral supernatants, retroviral vectors were transfected into Plat-E using FuGENE 6 (Promega) according to the manufacturer’s protocol. Viral supernatants were collected from culture medium at 48 h after transfection. Osteoblasts were infected with the retroviruses in the presence of 10 µg/mL polybrene (Sigma-Aldrich) for 6 h.

### 4.7. Statistical Analysis

All values are expressed as means ± standard deviation (SD). Statistical analyses were performed using two-tailed Student’s *t*-tests. *p*-values less than 0.05 were considered statistically significant.

## 5. Conclusions

The downregulation of *Runx2* mediated by 1,25(OH)_2_D_3_ in osteoblasts stimulates the transdifferentiation of osteoblasts to adipocytes, and this may partially contribute to decreased bone mass by decreasing bone formation and increasing bone resorption.

## Abbreviations

1,25(OH)_2_D_3_	1,25-Dihydroxyvitamin D_3_
ALP	Alkaline phosphatase
BSP	Bone sialoprotein
CEBP-α	CCAAT/enhancer binding protein-α
PPAR-γ	Peroxisome proliferator-activated receptor-γ
PPi	Pyrophosphate
VDR	Vitamin D receptor
VDRE	Vitamin D response element
